# Moral Disengagement, Dark Triad and Face Mask Wearing during the COVID-19 Pandemic

**DOI:** 10.3390/ejihpe12090090

**Published:** 2022-09-02

**Authors:** Gina Chávez-Ventura, Henry Santa-Cruz-Espinoza, Julio Domínguez-Vergara, Nancy Negreiros-Mora

**Affiliations:** 1 Institute for Research in Science and Technology, Universidad César Vallejo, Trujillo 13009, Peru; 2 Professional School of Psychology, Universidad Autónoma del Perú, Lima 15842, Peru; 3 Research Direction, Universidad Tecnológica del Perú, Lima 15046, Peru; 4 Professional Career of Psychology, Universidad Privada del Norte, Lima 15314, Peru

**Keywords:** Machiavellianism, psychopathy, narcissism, personality, face mask

## Abstract

Not wearing a face mask, despite the sanitary recommendation, represented a public health risk during the COVID-19 pandemic. For this reason, the aim of the study was to determine the mediating role of moral disengagement in the relationship between the dark triad and face mask wearing during the second wave of the pandemic. We worked with a sample made up of 534 adults, who were administered the Dirty Dozen Dark test, the Moral Disengagement Mechanisms Scale and a questionnaire on the frequency of use of face masks. The results showed that moral disengagement mediates the effect of each trait of the dark triad (Machiavellianism, psychopathy and narcissism) on the use of face masks. It is concluded that those who possess any of the dark personality traits morally disengage in order not to use a face mask, exercising a reckless behavior of the possible contagion of COVID-19 to others.

## 1. Introduction

The COVID-19 disease, because of insufficient scientific information on how to prevent it [1], quickly spread and became a pandemic [2]. For this reason, in order to avoid contagion, different governments adopted provisions that limited social interaction [3], such as the use of face masks covering the nose and mouth. Failure to comply with these measures meant exposing not only one’s own health to the risk of contagion or reinfection, but also that of family members and strangers [4].

Several studies have highlighted the factors associated with COVID-19 preventive behaviors. Among them, it has been reported that those with greater kindness [5,6], greater risk aversion or greater self-control or need for cognition, complied with the measures of mask use and social distancing [7]. Other research, on the other hand, has focused on dark personality traits, characterized by lack of empathy or social insensitivity [8]. Among them, the dark triad is traditionally investigated [9]: Machiavellianism, narcissism and psychopathy, which includes subclinical samples present in a larger range among the population at large, as opposed to clinical samples, which are under clinical or forensic supervision [10].

Machiavellianism is a personality trait characterized by manipulation [10,11], cynicism [10] and strategic thinking [12,13]. Those who present this trait have the cognitive ability to exploit others and plan to obtain rewards for their own benefit under situations of risk and uncertainty [14]; they are prone to misrepresent information, do not feel guilty about it and rationalize their actions [15]. This trait may develop in adulthood in the context of modern societies, characterized by greater stability, low mortality, higher income, economic freedom and education [16].

Narcissism, on the other hand, is characterized by feelings of grandiosity and vulnerability, in the search for self-affirmation [10]. It presents with the belief of being entitled to special treatment, having a need to receive admiration and recognition and lacking empathy [17], and is positively associated with well-being, unlike Machiavellianism and psychopathy, which are negatively related to well-being [18]. Subclinical narcissism is less severe than clinical narcissism and does not meet all the criteria to be diagnosed as a personality disorder [19]. Its typification varies and, in this regard, one study reports that it includes the factors of extraversion, antagonism and neuroticism, of which the least empathetic is the antagonist [20]. Another investigation classifies nonclinical narcissism into grandiose and vulnerable, and finds evidence in favor that emotional neglect occurring in childhood is associated with vulnerable narcissism and that parental overvaluation is linked to grandiose narcissism [17].

The last trait of the dark triad is subclinical psychopathy, characterized by low levels of kindness and responsibility [10], recklessness, impulsive vindictiveness [11] and disrespect for rules and authority [21]. As a clinical trait, it is linked to high rates of crime, recidivism and resistance to treatment [22]. It is the trait with the highest heritability [16,23] that is not static because it changes over time in interaction with age and context [22], in which family violence experiences predispose it [22,23].

The similarities among the traits of the dark triad are the tendency to be maladaptive, insensitive, selfish, malevolent [10] and determined [11]. Nevertheless, researchers have found that Machiavellianism and psychopathy share characteristics, independent of narcissism, and form a dark dyad characterized by impulsive revenge, selfishness and insensitivity, manipulation, recklessness, sensation seeking, distrust and a lack of morality [11]. Likewise, they have in common a dark personality core, which is especially explained by genetic components [21].

Research on the dark personality traits in the context of COVID-19 shows contradictory findings. On the one hand, it has been reported that it does not predict social distancing and that narcissists provided social support during the pandemic [24], whereas other studies found that dark personality traits predicted the intention to expose others to risk [5], to breach restrictions for virus spread [6,25,26] and to oppose vaccination [27].

From the social cognitive approach, refraining from transgressive actions, as well as engaging in prosocial actions, involve moral agency, which encompasses self-regulatory mechanisms rooted in moral self-sanctions, aligned to moral standards [28,29]. However, in contrast to moral control, moral disengagement is a psychosocial mechanism that occurs when people disengage with moral self-sanctions through justifications or reasoning that make their harmful behavior acceptable while maintaining their self-esteem [29]. It tends to occur gradually and becomes routine without a process of reflection; it even develops from childhood and is characterized by low feelings of guilt, low prosocial behavior and ruminations of perceived grievances or harms and vindictive retaliation, which elevates aggression and antisocial behavior [28,29].

There are eight mechanisms of moral disengagement, which lead to eliminating self-censorship and taking pride in harmful actions [28,29]: (a) moral justification involves cognitively redefining harmful actions so that they appear “socially approved”; (b) euphemistic labeling is used in order to cover up harmful behavior with “harmless” language devoid of humanity; (c) advantageous comparisons allow harmful behavior to appear good, depending on the fact or behavior to which it is compared, or on the basis of utilitarian standards; (d) minimizing, ignoring, distorting or disbelieving the harmful consequences, and this is facilitated when such effects are not visible and if the victims are not known; (e) blurring of moral responsibility when this falls on the group and not on a personal level; (f) displacing of responsibility, where the effects of the conduct are not assumed personally but “done” by a legitimate authority so that people do not feel responsible for their perpetrated harmful actions; (g) dehumanization, where the victims are stripped of their human qualities; (h) attributing blame to adversaries or to life circumstances or to the victim himself for provoking the mistreatment of himself; in that way, the aggressors perceive themselves as victims forced to transgress by circumstances or wrongdoers.

Moral disengagement is a predictor of criminal behavior that develops in the interaction of personal, behavioral, and social influences [28,29]. Proof of this is that moral disengagement, together with the dark triad, are directly related to antisocial behaviors in adolescents [30]. The dark triad tends to lead to moral disengagement and to the exercising of relational aggression manifested in joy at the suffering of others [31]. Additionally, high moral disengagement is associated with the perpetration of cyberbullying [32], especially in adolescents with high anger rumination [33] or in college students with insensitive–unemotional traits [34].

Although no reports of moral disengagement in the context of the COVID-19 pandemic have been found, one study showed that belief in COVID-19 conspiracy theories decreases trust in institutions, affects compliance with social distancing and reduces social engagement [35]. Different conspiracy theories are compatible with different moral disengagement arguments (e.g., COVID-19 “would be the result of secretive actions by those in power” or “has not been proven to exist”). In this regard, people with Machiavellianism or psychopathy, to a greater extent than collective narcissism, are more likely to hold and spread general conspiracy beliefs as a result of their attempt to manipulate others, whereas collective narcissism showed associations with conspiratorial beliefs specifically about COVID-19 [36].

In the scenario of the second wave of the COVID-19 pandemic and before possible future scenarios of new pandemics, it is pertinent to explain the behavior of not following preventive norms, such as the use of face masks, since it is of interest to public health. In addition, it will make it possible to fill a gap in the knowledge, as well as to provide information that will make it possible to carry out public health prevention actions. For this reason, the aim of the study is to determine the mediating role of moral disengagement in the relationship between the dark triad and wearing a face mask during the COVID-19 pandemic in Peruvian adults. In this regard, with the available evidence, and considering the characteristics of the traits of the dark triad, it is presumed that: (a) the manipulation of others and disinterest in others, typical of Machiavellianism, may lead to the development of arguments of moral disconnection (compatible with conspiracy theories) that justify the non-use of face masks during the pandemic; (b) people with narcissism, with a sense of grandiosity, may elaborate reasons for moral disengagement as they perceive themselves invulnerable to the disease and may resist the use of face masks that affect their image and self-promotion; (c) psychopathy may be prone to lead to moral disengagement to justify the non-use of the mask, as a reckless and vindictive mechanism. Based on what has been stated above, the following hypothesis is formulated: the dark triad influences the non-use of face masks mediated by moral disengagement.

## 2. Materials and Methods

### 2.1. Study Design and Procedure

The design was cross-sectional explanatory with observable variables [37], considering that it measures the potential effect of mediating variables. Prior to the execution of the study, the ethics committee of the Universidad Autónoma del Perú approved the research project (protocol code 0028, 4 June 2021). Subsequently, the target sample was informed online of the purpose of the study, what their participation would consist of, the estimated time, the anonymous and voluntary nature of the study, the possibility of withdrawing at any time without consequences and the means of contacting the researchers in case they had any doubts related to the study. Those who agreed chose the YES option on the informed consent form. The evaluation was conducted between September and December 2021, during the second wave of the pandemic. The participants completed an online form using the respondent-driven sampling technique [38] and the linkage was through virtual platforms (WhatsApp and Facebook), having a scope of 3 regions of Peru (Lima, La Libertad and Piura).

### 2.2. Participants

The sample, selected by non-probabilistic sampling, consisted of 534 people over 18 years of age who resided in Peru, had internet access and gave informed consent for the study. The participants were mostly women (392, 73.4%), with higher education (395, 74%) followed by technicians (86, 16%), with an average age of 26.4 years (SD = 9.3). Most of the participants in the study had not been ill with COVID-19 (354, 66.3%) and had no family members who had died from COVID-19 (346, 64.8%).

### 2.3. Measurement

#### 2.3.1. Dirty Dozen Dark Triad

Dirty Dozen Dark Triad (DDT) is a self-report questionnaire of the dark triad, created by Jonason and Webster [39] and consisting of the subscales of Machiavellianism, psychopathy and narcissism. It has a 5-point Likert format and has 12 items. For this study, psychometric validation was performed by means of a confirmatory factor analysis based on the three-factor related model [40] and obtained favorable indices (X^2^/gl = 2.9; CFI = 0.92; RMSEA = 0.06 IC90 [0.05–0.07]; SRMR= 0.05). Reliability was calculated with the omega coefficient, where the Machiavellianism (ω = 0.76), psychoticism (ω = 0.73) and narcissism (ω = 0.77) dimensions obtained values above 0.70.

#### 2.3.2. Moral Disengagement Scale

Moral Disengagement Scale (MDS) measures the psychological mechanisms that justify immoral actions without the constraint of negative self-sanction. The Bandura et al. scale [41], a 5-point Likert-type scale, is composed of 32 items. The psychometric findings for this study were obtained under the model of Rubio-Garay et al. [42]. Ten participants from the target population assessed the clarity of the items and, with their suggestions, three items were modified (7, 8 and 10). In validity, the model of one general second-order factor and three first-order factors shows a good fit for this study (χ2 = 30.1; RMSEA = 0.021; CFI = 0.980; TLI = 0.908; SRMR = 0.023). The factors obtained were: disengagement by depersonalization (including dehumanization and attribution of blame), disengagement by irresponsibility (including advantageous comparison, displacement of responsibility and diffusion of responsibility) and disengagement by rationalization (including moral justification, euphemistic labeling and distortion of consequences). The reliability calculation, with the omega coefficient, was acceptable for factors I (ω = 0.63), II (ω = 0.66) and III (ω = 0.70), and good for the general factor of moral disengagement (ω = 0.83).

#### 2.3.3. Face Mask Wearing

To measure face mask wearing, the following questions were asked: “In the last two weeks, how often have you worn a face mask when you leave your home?”; “In the last two weeks, how often have you made sure that the mask completely covers your nose and mouth?” and “Did you change the mask when the recommended wearing time was over?”. The response alternatives were 6-point Likert-type. In this study, the reliability by Cronbach′s Alpha is 0.70.

### 2.4. Statistical Analysis

The IBM SPSS Statistics 25 package was used and the mediation analysis was performed using the Macro PROCESS program for SPSS [43]. Descriptive statistics and correlations between variables were calculated, of which statistical significance was presented and the effect size was interpreted according to values of 0.10 for small, 0.30 medium and 0.50 large magnitude [44].

The calculation of the indirect effect of the mediation model [45] was considered and a bootstrapping of 10,000 simulations was used to determine the mediating effect with a 95% confidence interval. The significant effect was calculated by considering confidence interval values that were not on either side of zero [43].

## 3. Results

The findings show statistically significant correlations with a medium effect size between moral disengagement and traits of Machiavellianism and psychopathy. In addition, narcissism has a statistically significant small magnitude correlation with moral disengagement (Table 1).

**Figure 1:**
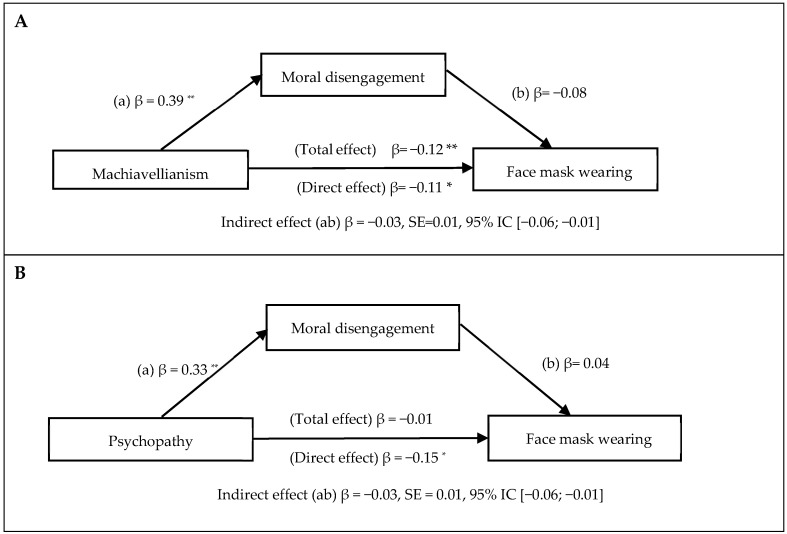

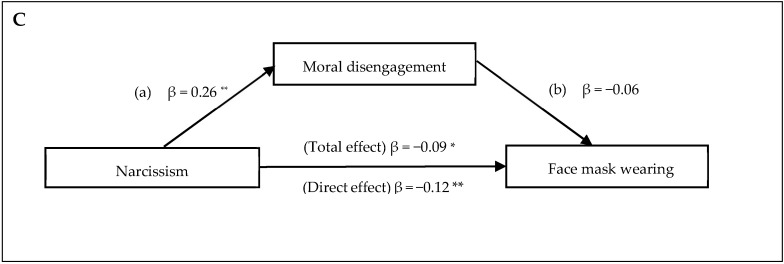
Models of mediation of moral disengagement in the relationship between the dark triad (machiavellianism [Panel **A**], psychopathy [Panel **B**], narcissism [Panel **C**]) and the use of masks. Routes a and b indicate the indirect effect of the dark triad on the use of masks, through moral disengagement. * *p <* 0.05, ** *p* < 0.01 (two tails).

Moreover, three explanatory models are presented where the indirect effect of the mediational analysis shows the confidence interval in which zero is not included, so that moral disengagement has a mediating effect on the relationship of Machiavellianism (Figure 1A), psychopathy (Figure 1B) and narcissism (Figure 1C) with the use of face masks in Peruvian adults.

## 4. Discussion

The findings show that moral disengagement is related to Machiavellianism, psychopathy and narcissism; furthermore, all three traits were mediated by moral disengagement in their effect on the use of face masks during the second wave of the COVID-19 pandemic. In other words, in the dark triad, moral disengagement acts as a self-regulating mechanism to exclude moral self-censorship and justify the non-use of the mask, despite the fact that it represents reckless behavior because, in the context of the pandemic, it spreads the disease, generates a risk of mortality, and risks the collapse of health services and the restriction measures that impact the national economy.

In the study, moral disengagement was reported to be a partial mediator in the relationship between narcissism and the non-use of face masks. People with a narcissistic trait, characterized by feelings of superiority and beliefs of inferiority of others [46], justify with arguments of depersonalization, irresponsibility and rationalization, the non-use of face masks, which threatens their own health and that of others. This finding could be attributed to the fact that people with narcissism, who have developed the antagonistic facet, tend to use masks less and disregard social distancing during the COVID-19 pandemic [47]. In addition, it could be due to their tendency towards self-promotion [48] and status seeking [46], so they may refuse to wear masks as they perceive them as unaesthetic.

In this regard, one report indicates that people with collective narcissism, who possess supervalued beliefs of the superiority of their group, tend to believe that they will not get sick from COVID-19 and report better physical health [49]. Thus, they may appear to others as invulnerable to the possible threat of COVID-19 contagion, denying—consequently—the possibility of contagion to others. Moreover, by believing in the conspiracy theories of COVID-19 [36], they develop arguments of moral disengagement, such as minimizing their own responsibility by attributing the contagion to people who leave their homes to expose themselves to the virus; and, by not observing the immediate consequences of a possible contagion of the disease, it is easier to pass the blame to third parties.

Conversely, narcissism was found to be positively related to self-protection, illness avoidance, group affiliation, and caring for relatives [50]. This discrepancy is possibly due to the fact that the narcissistic participants may have had more developed neuroticism or extroversion factors than the antagonistic factor; an aspect that has not been taken into consideration in this research, and, therefore, constitutes a limitation.

A finding similar to that of people with narcissism was obtained in those with the trait of Machiavellianism. Machiavellianism is also partially mediated by moral disengagement, in relation to the non-use of masks. Those who present this trait, in their eagerness to manipulate the interests of others in order to achieve their own purposes [50] and show disregard for morality through selflessness [40], adhere to conspiracy theories [36,51] or elaborate beliefs that justify cynicism and irresponsibility by eliminating self-censorship, which influences their behavior of not wearing face masks. In this way, this is in agreement with a study where Machiavellianism “justifies” their financial misbehavior (15); moreover, it is negatively associated with group affiliation and kinship care [49], although the mediating variable of moral disengagement was not considered in that report.

According to the above findings, the third trait of the dark triad, subclinical psychopathy, influences the non-use of face masks, partially mediated by moral disengagement. In that sense, people with psychopathy, characterized by high disinhibition, insensitive aggression and low impulse control [52,53] are morally disengaged and do not use face masks that prevent COVID-19 contagion. This finding is consistent with a study where it was found that people with higher psychopathy tend towards relational aggression and enjoy the suffering of others, mediated by moral disengagement [31] and even tend towards intentionally placing others at the risk of COVID-19 infection [5] and spreading conspiracy theories [36,51]. Consistent with the antecedents, subclinical psychopathy is negatively associated with self-protection, social affiliation, caring for relatives [50] and adherence to COVID-19 restraint measures [25,26,54].

The findings obtained add to the evidence that moral disengagement acts as a socio-cognitive strategy that justifies morally unacceptable actions to perpetrate harm to others [34,55,56], even at the risk of their own health, in the context of the pandemic. These results are confined, in particular, to young people with higher education, which leaves gaps that require further research, because there is evidence that parents tend to assume more protective behaviors regarding the pandemic [57]. In addition, the majority of participants were women, which represents a bias for the research because there is evidence of sex differences in dark triad traits [16]. Therefore, it is suggested to future researchers to consider sex as a control variable.

One of the limitations of the study was that it collected the information with self-report scales, which may contain some proportion of social desirability. Another aspect that could be overcome in future research would be to measure each dark personality trait independently so that greater precision can be obtained in the results by knowing the factors of each one. In addition, it would be helpful to carry out longitudinal studies to determine the causal relationships between the variables.

The research did not cover the sadism trait [58], which is a dark personality trait, so future research could consider it. In addition, the external validity of the study could have been affected by having worked with a non-probability sampling. On the other hand, the time unit in which the instruments were applied was a limiting factor because the time elapsed after the evaluation brought with it pandemic fatigue and milder forms of the disease, which have not been considered in the study. Moreover, having been vaccinated or having been infected with COVID-19 could be intervening variables in the behaviors of reducing protective measures against the disease, which have not been taken into account.

The findings show that dark personality traits explain the reckless behavior of the non-use of masks during the pandemic, mediated by moral disengagement. For this reason, at a practical level, in the context of the pandemic, it is suggested to promote prosocial messages [59], awareness raising and emotional recognition actions [60], which contribute to the reduction in unempathetic behaviors and to the favoring of preventive measures. Likewise, at the individual level, it is recommended not to confront those who do not use face masks because this could expose oneself to a reckless reaction from the transgressor [61] due to their personality characteristics. It is therefore advisable for health professionals to carefully observe their behaviour so as to not be misled by their arguments of moral disengagement, nor by the tendency of dark personalities to have a socially attractive appearance [62].

Finally, as a public health measure at the preventive level, it is relevant to promote the formation of personality traits that adequately regulate emotions, and, although this study focused on already structured personality, it is possible to consider the available research [17,22,23] for primary prevention. It is necessary for health professionals to promote healthy bonds from an early age so that from childhood, emotions are adequately regulated, empathy is developed and responsibility for one’s own actions is assumed, aligned with moral standards. For this purpose, serial dramas, which express people’s fears, efforts and expectations, can be used as resources that contribute to the reader’s personal change; they are based on the integration of social cognitive theory, the translational and implemental model and the social diffusion model [29]. As Bandura points out, it is necessary to stop dehumanizing practices, to stop justifying harmful actions towards others and develop humanity based on shared relational experiences, where one’s own well-being is linked to that of others [29].

## 5. Conclusions

Dark personality traits (Machiavellianism, narcissism and psychopathy) are morally disengaged to avoid self-censorship for not wearing masks, despite their mandatory use as a health measure, in the context of the second wave of the COVID-19 pandemic. These findings help to explain the non-compliance with preventive behavior and the rapid spread of the disease, making it imperative to consider the proposed measures, especially in view of possible future health emergency scenarios.

## Figures and Tables

**Table 1:** Descriptive measures and correlations of the study variables (*n* = 534).

Variables	M	SD	Min	Max	1a	1b	1c	2	3
1. Dark triad									
1a. Machiavellianism	6.77	2.53	4	16	1	0.343 **	0.411 **	0.388 **	−0.119 **
1b. Psychopathy	7.66	2.79	4	18		1	0.229 **	0.334 **	−0.008
1c. Narcissism	9.65	3.39	4	20			1	0.258 **	−0.095 *
2. Moral disengagement	52.32	14.38	32	116				1	−0.137 **
3. Use of face masks	13.89	1.88	2	18					1

* *p <* 0.05, ** *p* < 0.01 (two tails); M = median, SD = standard deviation, Min = minimum, Max = maximum.

## Data Availability

The data presented are available upon request to the corresponding author for academic and research use.
